# Endothelial complications after allogeneic stem cell transplantation in patients with pretransplant resolved COVID-19

**DOI:** 10.1038/s41409-022-01660-3

**Published:** 2022-04-20

**Authors:** Christian Niederwieser, Bodo Weber, Mirjam Reichard, Nico Gagelmann, Salem Ajib, Vera Schlipfenbacher, Zhen Zeng, Fabian Lang, Dietlinde Janson, Christine Wolschke, Francis Ayuk, Gesine Bug, Nicolaus Kröger

**Affiliations:** 1 University Medical Center Hamburg Eppendorf UKE, Hamburg, Germany; 2 Department of Medicine, Hematology and Oncology, Goethe University, Frankfurt/Main, Germany

**Keywords:** Infectious diseases, Signs and symptoms, Epidemiology

## To the Editor:

Given the considerable overlap between endothelial dysfunction caused by the coronavirus disease 2019 (COVID-19)-associated endotheliitis and the one observed after allogeneic HSCT such as veno-occlusive disease (VOD), transplant associated-thrombotic microangiopathy (TA-TMA), idiopathic pneumonia syndrome (IPS) and graft-versus-host disease (GvHD) we analyzed long- term outcomes of patients who received allogeneic-HSCT with recovered COVID-19 [1, 2]. Favorable short-term outcomes after HSCT were previously reported by our group [3].

A total of 14 patients with a history of resolved COVID-19 were transplanted at the University Medical Center Hamburg Eppendorf UKE (*n* = 9) and Department of Medicine of Goethe University Frankfurt (*n* = 5) and provided informed consent for data collection and analysis. Patients were 57.1% female and 42.9% male with a median age of 56.5 (range 33–69) years (Table 1). COVID-19 was diagnosed between February 2020 and June 2021 by polymerase chain reaction (PCR) a median of 26 (−99 - 134) days before or after induction chemotherapy for advanced or high-risk acute myeloid (AML), lymphoblastic leukemia (ALL) and blast crisis of CML. During COVID-19, 11 patients (79%) developed lung infiltrates, six patients (43%) required ICU admission and two were treated with casirivimab/imdevimab (H8) or bamlanivimab (Fr2, Table 1). All patients recovered after median 47.5 (11–70) days after diagnosis of COVID-19 and were transplanted a median of 94 (35–136) days after COVID-19 resolution in CR1 (*n* = 8), PR or CR3 (*n* = 2) of high risk AML, high risk ALL (*n* = 3) or second chronic phase after CML blast crisis (*n* = 1). Donors were matched related (*n* = 3) or unrelated (*n* = 4), haploidentical related (*n* = 4) or mismatched unrelated (*n* = 3). Conditioning was myeloablative (MAC; *n* = 6), of reduced intensity (RIC; *n* = 7) and of reduced toxicity (RTC; *n* = 1). All patients received fludarabine in combination with fractionated total body irradiation (TBI; 8 or 12 Gy, *n* = 7), thiotepa/busulfan (*n* = 3), melphalan (*n* = 3) or treosulfan (*n* = 1). GvHD prophylaxis was performed with anti-thymocyte globulin (ATG) followed by cylosporine and mycofenolate mofetil (MMF) in 10 patients and with post-transplant cyclophosphamide (PT-CY) and tacrolimus/MMF in the haploidentical (4) and in one mismatched unrelated HSCT. Ursodiol as prophylaxis was given to all patients transplanted in Frankfurt/Main. TA-TMA was defined as previously described [4] and VOD diagnosed according to Seattle criteria [5]. Differences between groups were analyzed with the Fisher’s exact test.

**Table 1:** Characteristics of patients recovered pretransplant from COVID-19 and diagnosis of endothelial complications.

*H* Hamburg, *Fr* Frankfurt/Main, *M* male, *F* female, *AML* acute myeloid leukemia, *t* therapy related, *ALL* acute lymphoblastic leukemia, *CML BC* blast crisis CML, *chr.Ph.CML* chronic phase CML, *n.a.* not available, *ven/aza* venetoclax/azacytidine, *7 + 3* according to [9]; *mido* midostaurin, *GO* gentuzumab ozogamicin, *HAM* high-dose cytosine arabinoside (HDAraC) and mitoxantrone, *mito-FLAG* fludarabine AraC mitoxantrone granulocyte colony-stimulating factor, *GMALL* German ALL protocol, *blina* binatumumab, *OSHO* East German Study Group protocol, *EWALL* European Working Group on Adult ALL, *CT* computed tomography, *pneum* pneumonia, *pneum*^*§§*^ bilateral pneumonia, *peri* pericarditis, *RI* respiratory insufficiency, *ARF* acute renal failure, *CRBSI* catheter-related blood stream infection, *DVT* deep venous thrombosis, *O2* oxygen, *ICU* intensive care unit.

*Intubated; *HSCT* hematopoietic stem cell transplantation.

**Diagnosis COVID-HSCT days;* L* lopinavir, *RIT* ritonavir, *P* prednison, *R* remdesivir, *dexa* dexamethason, *casi* casirivimab, *serum* convalenscence serum, *imde* imdevimab, *bamla* bamlanivimab, *CR* complete remission, *MAC* myeloablative conditioning, *RIC* reduced intensity conditioning, *RTC* reduced toxicity conditioning, *Gy* Gray, *TBF* thiotepa busulfan fludarabine, *flu* fludarabine, *mel* melphalan, *TBI* total body irradiation, *treo* treosulfan, *ATG* antithymocyte globulin, *PT-CY* post-transplant cyclophosphamide; *MRD* matched related donor, *MUD* matched unrelated donor, *MMUD* mismatched unrelated donor, *haplo* haploidentical related donor, *TA-TMA* transplant associated thrombotic microangiopathy, *VOD* venoocclusive disease, *defib* defibrotide, *ecu* eculizumab, *n.ap.* not applicable, *ple* pleural effusion, *peri* pericardial effusion, *asc* ascites.

After a median follow-up of 221 (69–492) days, 11 (79%) of the 14 patients are alive. One patient died on day +146 from complications following AML relapse, one from cardio-pulmonary insufficiency following fungal infection (d +208) and one from not further specified liver failure (d +179). Three patients developed VOD, TA-TMA or both, all of them associated with polyserositis, at a median of day +67.5 (9–242) and are still alive a median of 451 (221–492) days after HSCT. Additional four patients had serositis without clinical signs of TA-TMA or VOD.

Patients with TA-TMA/VOD were exclusively female transplanted from a male mismatched unrelated or family donor (*p* = 0.01) in comparison to patients without TA-TMA/VOD. In addition, seven out of the 14 patients developed pleural, pericardial effusion and ascites after HSCT. In contrast, no differences in age, interval diagnosis leukemia to COVID-19, COVID-19 duration, interval from COVID-19 diagnosis to HSCT, conditioning including TBI, conditioning intensity and GvHD prophylaxis were detected between the non- and TA-TMA/VOD patients (Table 1; p = n.s.). In a subgroup of patients (*n* = 9) median peak levels of acute phase proteins such as ferritin (3440 vs 3817 µg/l), IL-6 (323.45 vs 767.8 ng/l), procalcitonin (PCT; 2.48 vs 1.87 µg/l) and C-reactive protein (CRP; 185 vs 256 mg/l) during COVID-19 were found to be not significantly different in patients with as compared to those without endothelial damage.

One 69 years old female patient (H1) with AML achieved CR1 after induction with azacytidine and venetoclax and developed on the day of leukemia diagnosis pulmonary COVID-19 requiring mechanical ventilation. Peak ferritin reached 2875 µg/l, IL-6 538 ng/l, PCT 5 µg/l and CRP 361 mg/l. She was tested PCR negative after 12 days. Haploidentical HSCT was performed 106 days after COVID-19 following a conditioning regimen with thiotepa, busulfan, fludarabine and ATG. Tacrolimus/MMF in combination with PT-CY were administered for GvHD prophylaxis. Cytomegalovirus (CMV) status was negative in both donor and recipient. After engraftment on day +17, she developed histologically (renal biopsy) confirmed TA-TMA eight months after HSCT. CMV, urogenital and clostridium difficile infections may have triggered TA-TMA. The patient recovered from TA-TMA after steroids and antiviral therapy and deteriorated again in a subsequent CMV reactivation. The newly diagnosed polyserositis was treated with prednisone. The patient is alive 492 days after HSCT.

One patient (H4) with ALL was treated according to GMALL (German ALL) protocol and three courses of blinatumomab and recovered from COVID-19 after treatment with convalescent serum with peak levels of ferritin 4006 µg/l, IL-6 109 ng/l, PCT 0 µg/l and CRP of 85 mg/l. Duration of infection was 56 days. After conditioning regimen of TBI and fludarabine a haplo-identical HSCT from a male, CMV positive donor (recipient positive) was performed. For GvHD prophylaxis, immunosuppression with tacrolimus/MMF and PT-CY was given. Engraftment was observed on day +21 and on day +59, CMV reactivation, BK cystitis, polyserositis and ascites were diagnosed. Pathological liver enzymes and liver histology confirmed VOD, which was treated successfully with defibrotide. The patient is alive 451 days post-HSCT.

One patient (Fr2) with AML had disease persistence following 7 + 3+midostaurin and obtained CR1 after high dose cytarabine and mitoxantrone (HAM) followed by gilteritinib. COVID-19 was detected by PCR 104 days after diagnosis and persisted for 70 days despite convalescent serum, dexamethasone, remdesivir and bamlanivimab. HSCT from a HLA-C antigen mismatched unrelated male and CMV concordant positive donor was performed 240 days after AML diagnosis and 136 days after COVID-19. In the immediate post-transplantation period, bacteremia with Enterococcus faecium was treated successfully with antibiotic combination. The patient was diagnosed with VOD (day +9) and treated successfully with defibrotide. On day +40, Epstein Barr Virus reactivation and human poliomavirus 1 - cystitis occurred. On day +64, the patient stabilized and gilteritinib maintenance was initiated. When she developed TA-TMA on day +76, she was switched from cyclosporine to everolimus. On day +81, acute GvHD of the gut was suspected which responded to steroids. Progressive kidney injury developed despite 6 doses of eculizumab starting on day +87, by ruxolitinib or daily plasma exchange starting on day +132. The patient remained on renal replacement therapy 221 days after HSCT.

The association of female patients transplanted from a mismatched male donor with endothelial damage and polyserositis was statistically significant despite the small sample size and needs further confirmation. Gender has shown to have an important role in immune response against COVID-19 and may provide a rationale for this observation [6]. Associations were found in addition with the presence of viral and bacterial infections. No association were found with older age, longer interval diagnosis—COVID-19, treatment of COVID-19, especially with convalescent serum and monoclonal antibodies (Table 1). In five of the six patients pleural effusions may be caused by endothelial dysfunction in the context of idiopathic pneumonia syndrome [7].

The small sample size, the lack of a control group and underdiagnosing endothelial damage may represent limitations of the study. Therefore, incidence of TA-TMA or VOD cannot be compared to that of patients transplanted without recovered COVID-19 [1, 8].

In summary, complications of endothelial damage was not associated with mortality, but with significant morbidity. Prophylaxis with endothelial-protective agents may represent a promising and rationale therapeutic strategy in female patients with mismatched or haploidentical HSCT from male donors and pretransplant COVID-19 history, especially if inflammation triggered by viral and bacterial infection is present. Complement inhibition for treatment of endothelial damage may be another approach to investigate.

Future research is needed in a larger group of patients to confirm our findings, identify new associations possibly missed in this small sample size and investigate prophylactic and treatment interventions.

## Data Availability

The datasets generated during and/or analyzed during the current study are available from the corresponding author on reasonable request.
